# The H_2_/CH_4_ ratio during serpentinization cannot reliably identify biological signatures

**DOI:** 10.1038/srep33821

**Published:** 2016-09-26

**Authors:** Ruifang Huang, Weidong Sun, Jinzhong Liu, Xing Ding, Shaobang Peng, Wenhuan Zhan

**Affiliations:** 1 Key Laboratory of Mineralogy and Metallogeny, Guangzhou Institute of Geochemistry, Chinese Academy of Sciences, 510640 Guangzhou, PR China; 2 Key Laboratory of Marginal Sea Geology, South China Sea Institute of Oceanology, Chinese Academy of Sciences, 510301 Guangzhou, PR China; 3 State Key Laboratory of Organic Geochemistry, Guangzhou Institute of Geochemistry, Chinese Academy of Sciences, 510640 Guangzhou, PR China; 4 State Key Laboratory of Isotope Geochemistry, Guangzhou Institute of Geochemistry, Chinese Academy of Sciences, 510640 Guangzhou, PR China; 5 Zhongshan Research Institute of Environmental Protection, 528400 Zhongshan, PR China

## Abstract

Serpentinization potentially contributes to the origin and evolution of life during early history of the Earth. Serpentinization produces molecular hydrogen (H_2_) that can be utilized by microorganisms to gain metabolic energy. Methane can be formed through reactions between molecular hydrogen and oxidized carbon (*e.g.*, carbon dioxide) or through biotic processes. A simple criterion, the H_2_/CH_4_ ratio, has been proposed to differentiate abiotic from biotic methane, with values approximately larger than 40 for abiotic methane and values of <40 for biotic methane. The definition of the criterion was based on two serpentinization experiments at 200 °C and 0.3 kbar. However, it is not clear whether the criterion is applicable at a wider range of temperatures. In this study, we performed sixteen experiments at 311–500 °C and 3.0 kbar using natural ground peridotite. Our results demonstrate that the H_2_/CH_4_ ratios strongly depend on temperature. At 311 °C and 3.0 kbar, the H_2_/CH_4_ ratios ranged from 58 to 2,120, much greater than the critical value of 40. By contrast, at 400–500 °C, the H_2_/CH_4_ ratios were much lower, ranging from 0.1 to 8.2. The results of this study suggest that the H_2_/CH_4_ ratios cannot reliably discriminate abiotic from biotic methane.

Serpentinization, a hydrothermal alteration of ultramafic rocks (typically peridotite and komatiite), occurs in a great variety of tectonic settings on the Earth, including the ocean floor, mid-ocean ridges and subduction zones^1^,^2^,^3^,^4^,^5^,^6^, and also on Mars^7^,^8^,^9^. Serpentinization dramatically modifies chemical and physical properties of oceanic lithosphere^10^,^11^,^12^,^13^,^14^,^15^,^16^. It induces a decrease in density and possibly an increase in volume^5^. A low degree of serpentinization (5–10%) could greatly weaken the strength of peridotite^10^. Serpentine, with a chemical formula of Mg_3_Si_2_O_5_(OH)_4_, can incorporate up to 13.5 wt% H_2_O and large quantities of fluid-mobile elements, such as Be, Ba and Cs^11^,^12^,^13^,^14^,^15^,^16^,^17^. In particular, serpentine can be stable at depths greater than 200 km^18^,^19^. Therefore, serpentine is an important chemical reservoir in subduction zones.

Serpentinization produces molecular hydrogen (H_2_), resulting from the oxidation of ferrous iron in olivine and pyroxene to ferric iron (Reaction (1)). Abiotic methane (CH_4_) can be derived from reactions between H_2_ and oxidized carbon (*e.g.*, carbon dioxide) through Fischer-Tropsch type (FTT) synthesis (Reaction (2)). Molecular hydrogen and methane support microbial communities in hydrothermal fields^20^,^21^,^22^,^23^,^24^,^25^,^26^,^27^. Methane may be produced biologically by methanogenic archaea^28^. The identification of abiotic and biotic methane is essential to understand ultramafic ecosystems, which potentially contribute to the origin and evolution of life during early history of Earth and possibly other terrestrial planets.
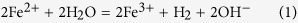

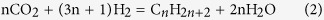


Traditionally, stable isotopes of carbon were used for identifying abiotic and biotic methane, because the δ^13^C values of abiotic methane differ largely from those of biotic methane. However, abiotic methane synthesized in the presence of elevated Fe-Ni alloys has δ^13^C values as depleted as those of biotic methane^29^,^30^. Recently, a simple criterion, the H_2_/CH_4_ ratio, was proposed to distinguish abiotic from biotic methane, based on two serpentinization experiments at 200 °C and 300 bars^27^. H_2_/CH_4_ ratios greater than approximately 40 mostly likely represent abiotic methane, and values less than 40 indicate biotic methane^27^. However, it is not clear whether the criterion is applicable at a wider temperature range. Temperature greatly influences H_2_ production during serpentinization^31^,^32^,^33^. The production of H_2_ reaches a maximum at ~300 °C^31^,^33^, whereas it largely decreases at temperatures higher than 350 °C^33^, possibly leading to a dramatic decrease in the H_2_/CH_4_ ratio. Previous experiments were primarily conducted at ≤300 °C^27^,^31^,^33^,^34^,^35^,^36^, and the temperature dependence of H_2_/CH_4_ ratios was poorly constrained.

In this study, we performed sixteen experiments at 311–500 °C and 3 kbar using natural ground peridotite with initial grain sizes of <30, 42–59, and 100–177 μm. The objectives of this study were (1) to investigate the temperature dependence of H_2_/CH_4_ ratios and (2) to explore whether H_2_/CH_4_ ratios can be used to identify abiotic and biotic methane at a wider range of temperatures.

## Results

### The H_2_/CH_4_ ratios

**Figure 1:**
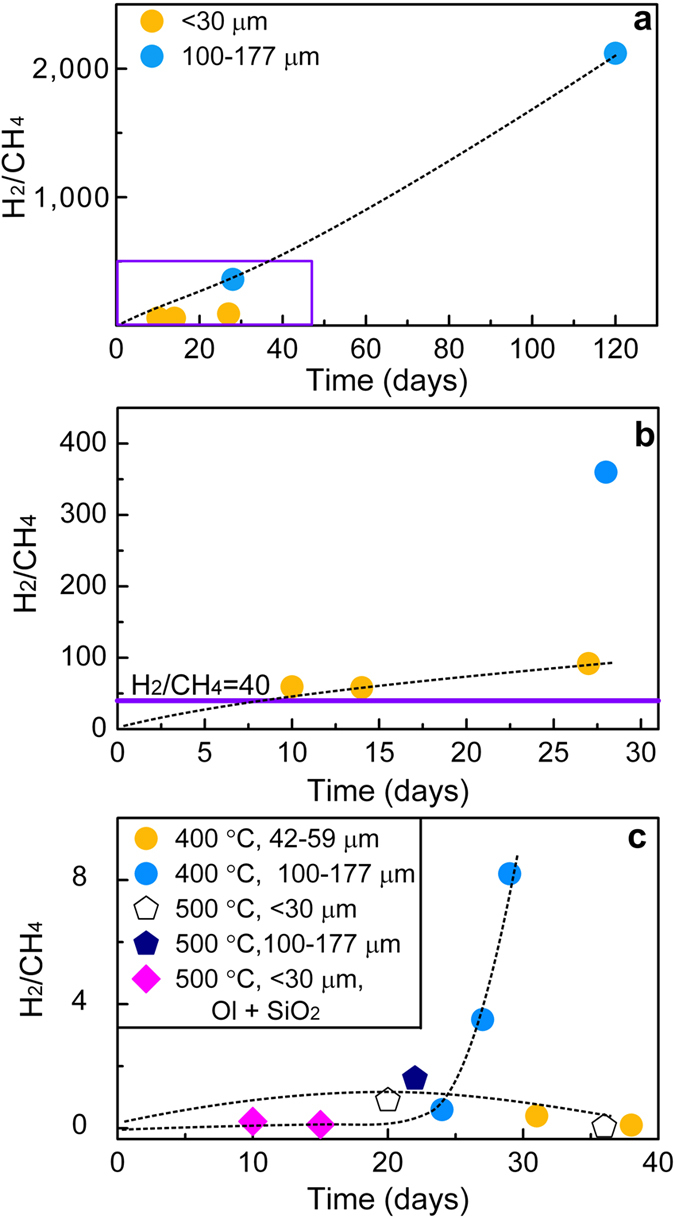
Plot of H_2_/CH_4_ ratios as a function of time (in days), showing strong temperature dependence. (**a**) 311 °C and 3.0 kbar. (**b**) An enlargement of the rectangle in (**a**). The critical number 40 is shown as a horizontal curve. (**c**) 400–500 °C and 3.0 kbar.

Molecular hydrogen, methane, ethane, and propane were formed. At 311 °C and 3.0 kbar, the H_2_/CH_4_ ratios ranged from 58 to 2,120, much higher than the critical value of 40 (Fig. 1a,b). The ratios increased as a function of time, implying that rates of H_2_ production are faster than the rates of CH_4_ formation. In experiments using peridotite with initial grain sizes <30 μm, the H_2_/CH_4_ ratios varied from 58 to 91. By contrast, for those using larger grain sizes (100–177 μm), the H_2_/CH_4_ ratios were much higher, from 360 to 2,120. At 400–500 °C and 3.0 kbar, the H_2_/CH_4_ ratios decreased greatly, 0.1–8.2 (Fig. 1c). In experiments at 500 °C and 3.0 kbar using peridotite with initial grain sizes of <30 μm, the H_2_/CH_4_ ratios increased during the first 20 days to a maximum value and then decreased slightly during the subsequent 16 days. This decrease suggests an increase of CH_4_ production (Table 1). A similar trend was also observed at 400 °C and 3.0 kbar with grain sizes of 42–59 μm, whereas it was not detected in experiments with larger grain sizes.

### Solid products

**Figure 2:**
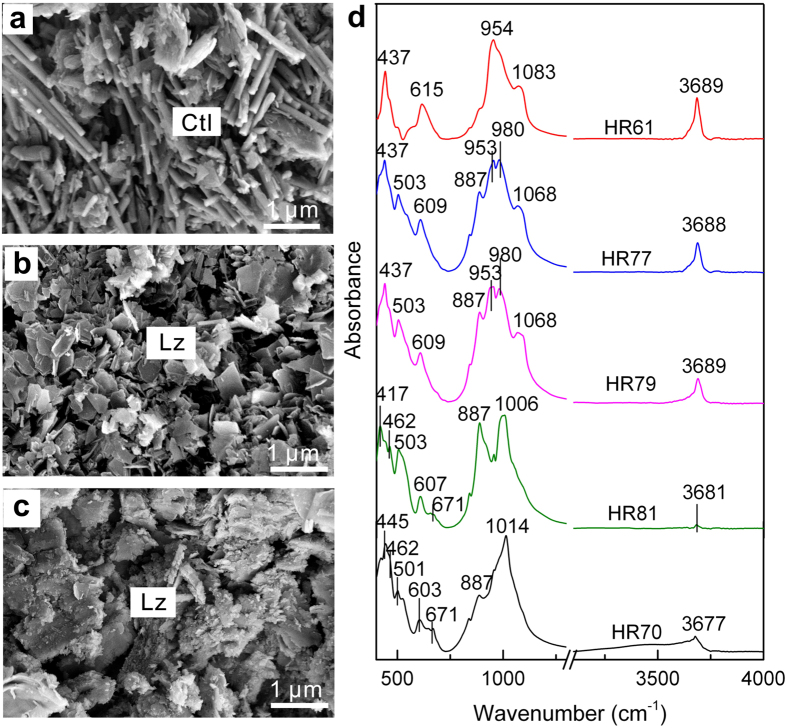
Identification of solid products by scanning electron microscope and Fourier transformed infrared spectroscopy analyses. (**a**) HR61, 311 °C and 3.1 kbar, with the formation of fibrous chrysotile (Ctl). (**b**) HR79, 400 °C and 3.0 kbar, with tabular shaped lizardite (Lz). (**c**) HR81, 500 °C and 3.3 kbar, with lizardite. **(d)** Infrared spectra of solid products. The spectra indicate that serpentine formed at 311–400 °C, whereas serpentine and talc were produced at 500 °C and 3.3 kbar. Talc was identified based on its stretching mode at 671 cm^−1^ for Si-O-Mg and a stretching vibration at 3677 cm^−1^ for the –OH group^42^.

At 311 °C and 3.0 kbar, the major secondary hydrous mineral was fibrous chrysotile (Fig. 2a), whereas tabular shaped lizardite formed at 400 °C and 3.0 kbar (Fig. 2b). Serpentine was identified based on infrared spectra with stretching modes at 954 and 1087 cm^−1^ for the Si-O group and a stretching vibration at 3686 cm^−1^ for the –OH group (Fig. 2d)^37^,^38^,^39^. Chemical compositions of secondary minerals in HR61 were provided in an experimental study^40^, consistent with compositions of serpentine^41^. At 500 °C and 3.0 kbar, the secondary hydrous minerals produced were talc and lizardite. Talc is characterized by a stretching mode at 671 cm^−1^ for Si-O-Mg and a stretching vibration at 3677 cm^−1^ for the –OH group (Fig. 2d)^42^.

## Discussion

**Figure 3:**
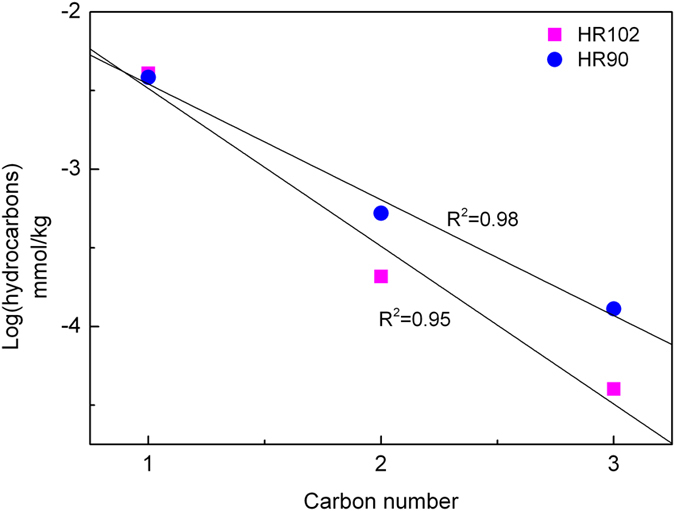
Schulz-Flory distribution of hydrocarbons produced in this study. The two solid lines show the least-squares fit of the data at 400 °C and 3.0 kbar (Table 1) using Origin 8.6 with correlation coefficients (R^2^).

The hydrocarbons produced in this study are probably abiotic, supported by the following evidence. First, blank experiments were performed at 311–500 °C and 3.0 kbar using peridotite loaded without any fluid. The quantities of H_2_ and hydrocarbons were below the detection limit of gas chromatograph after 27 days of reaction time. It suggests that hydrocarbons were not released from the decomposition of organic matter and long-chain hydrocarbons in peridotite^43^,^44^. Otherwise, it would result in highly elevated hydrocarbons. Moreover, the log of the n-alkane concentrations is linearly correlated with the carbon numbers (Fig. 3), which is consistent with the Schulz-Flory distribution predicted for FTT synthesis^31^. All these indicate that hydrocarbons were formed through reactions between H_2_ and dissolved carbon dioxide from the atmosphere in the starting fluid.

**Figure 4:**
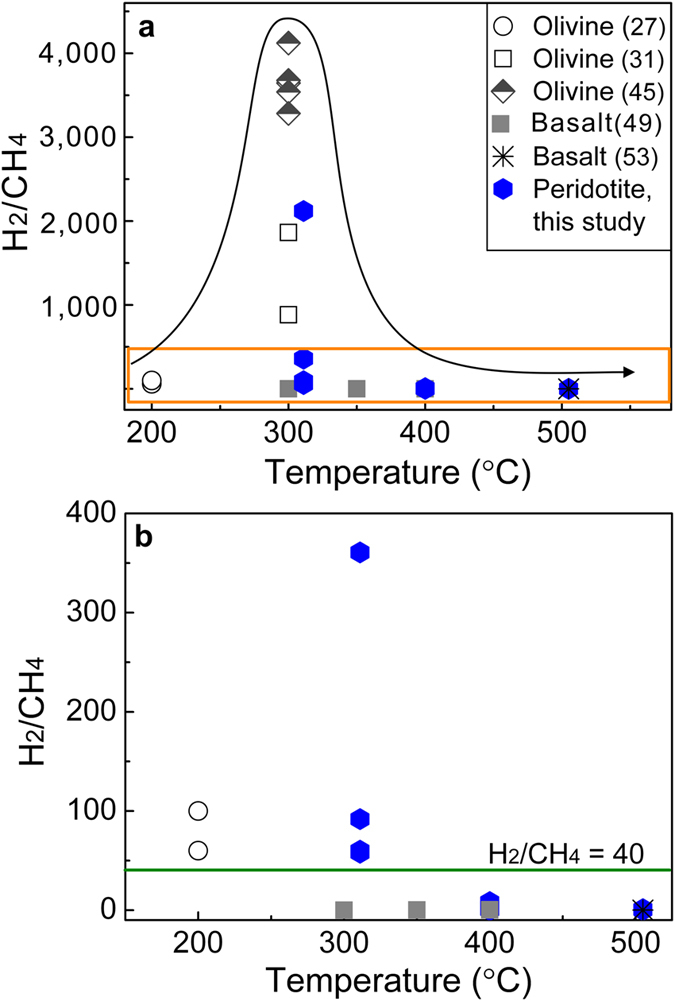
The H_2_/CH_4_ ratio as a function of temperature. (**a**) Comparisons of H_2_/CH_4_ ratios in this study (in blue) and those in previous work (in black)^27^,^31^,^45^,^49^,^53^. (**b**) An enlargement of the yellow rectangle in (**a**).

A plot of H_2_/CH_4_ ratios as a function of temperature is illustrated in Fig. 4, showing that the H_2_/CH_4_ ratios greatly depend on temperature. They reached their maximum values at ~300 °C, from 58 to 4,000 (Fig. 4a)^31^,^45^. By contrast, the values were much lower at 400–500 °C, much less than 40 (Fig. 4), resulting from the dramatic decrease in H_2_ production and increase in CH_4_ formation. The decrease in H_2_ production may be induced by very slow rates of olivine serpentinization at temperatures higher than 350 °C^46^,^47^,^48^, supported by infrared spectra of solid products with a sharp peak centered at 503 cm^−1^ for the Mg-O group of olivine and a weak band at 3677 cm^−1^ for the –OH group of talc (Fig. 2d). It suggests that H_2_ is mostly derived from orthopyroxene alteration. As indicated by experimental studies, the quantities of H_2_ produced during orthopyroxene alteration at >350 °C were one to two orders of magnitude less than those formed after olivine serpentinization at 300 °C^31^,^32^. Consequently, H_2_ production at 400–500 °C decreases greatly. By contrast, CH_4_ concentrations increased at higher temperatures (Table 1), which possibly results from sufficient Fe-Ni alloys that highly enhance CH_4_ production^29^.

Initial grain sizes of peridotite greatly influence the production of H_2_ and CH_4_, and the H_2_/CH_4_ ratios. Smaller grain sizes result in larger quantities of H_2_ and CH_4_ (Table 1). Grain sizes exert a strong influence on serpentinization rates, with smaller grain sizes for faster rates^48^. For experiments with the same run durations, peridotite with smaller grain sizes has larger reaction extents^48^. As suggested by an experimental study, the production of H_2_ showed a positive correlation with reaction extents of serpentinized peridotite^34^, and consequently smaller grain sizes result in more H_2_. Larger reaction extents possibly lead to the formation of more catalytic minerals (*e.g.*, Fe-Ni alloys), which could greatly enhance CH_4_ production^29^.

Run durations have great effects on H_2_/CH_4_ ratios (Fig. 1). At 311 °C and 3.0 kbar, the H_2_/CH_4_ ratios increased with longer time, implying that rates of H_2_ production are faster than rates of CH_4_ formation. By contrast, for experiments at 400–500 °C with smaller grain sizes (*e.g.*, <30 and 42–59 μm), the H_2_/CH_4_ ratios first increased to a maximum value, and then they decreased slightly during the subsequent reaction time (Fig. 1c). It implies that rates of CH_4_ production were slow at the onset of reactions, possibly resulting from insufficient catalytic minerals (*e.g.*, Fe-Ni alloys). When reactions proceeded, more catalytic minerals formed, which promote CH_4_ production, leading to a decrease in H_2_/CH_4_ ratios. By contrast, for experiments at 400 °C using peridotite with grain sizes of 100–177 μm, the H_2_/CH_4_ ratios increased with time, whereas their maximum values were not reached. It implies that longer time is needed for peridotite with larger grain sizes to achieve maximum H_2_/CH_4_ values.

Fluid compositions (*e.g.*, dissolved silica) may dramatically influence the H_2_/CH_4_ ratios. As indicated by an experimental study, basalt alteration at 300 °C produced H_2_ concentrations approximately two orders of magnitude less than those after peridotite serpentinization, resulting in very low H_2_/CH_4_ ratios, 0.04^49^. Consistently, fluids recharged from basalt-hosted hydrothermal fields have much lower H_2_/CH_4_ ratios than those from peridotite-hosted hydrothermal fields^50^. It is possibly because basalt alteration releases one to two orders of magnitude more dissolved silica into hydrothermal fluids^49^. Silica impedes the production of magnetite^51^, and consequently H_2_ production decreases greatly^52^. By contrast, for experiments at 400–500 °C, differences in H_2_ between basalt and peridotite hydration are much less significant^49^,^53^, leading to comparable H_2_/CH_4_ ratios (Fig. 4, Table 1).

As discussed above, the H_2_/CH_4_ ratios during serpentinization can be greatly influenced by many factors, including temperature, initial grain sizes of peridotite, run durations, and the dissolved silica in hydrothermal fluids. The H_2_/CH_4_ ratios of <40 can be achieved at temperatures higher than 350 °C or in the presence of silica, which may not necessarily represent biological signatures. In hydrothermal fields, peridotite commonly experiences a retrograde metamorphism, and serpentinization may occur at a wide range of temperatures^5^. It indicates that the production of H_2_ in hydrothermal fields can be greatly influenced by temperature. Additionally, high-temperature reactions (aside from serpentinization), microbial oxidation and sulphate reduction possibly affect H_2_ production^54^, and consequently the H_2_/CH_4_ ratios may be modified. All these indicate that the H_2_/CH_4_ ratios cannot reliably identify abiotic and biotic methane.

Interestingly, methane produced in this study has δ^13^C values larger than −30‰ (referenced to Pee Dee Belemnite, Table 1), consistent with isotopic compositions of abiotic methane^55^. By contrast, methane synthesized in the presence of elevated Fe-Ni alloys has very depleted δ^13^C values, much lower than −30‰^29^,^30^. Iron-Ni alloys are accessory minerals in serpentinites, typically less than 0.5%. Therefore, experiments conducted using elevated Fe-Ni alloy may not represent natural hydrothermal systems. As reported in an experimental study, δ^13^C values of methane greater than −30‰ was detected in one experiment, whereas in the other experiment under the same condition, methane had δ^13^C values lower than −30‰^56^. In particular, the δ^13^C values of methane became more depleted with longer time^56^. Therefore, it is not clear whether stable isotopes of carbon can effectively identify abiotic and biotic methane.

## Materials and Methods

A non-altered peridotite was reacted with NaCl fluid (0.5 mol/L dissolved NaCl; ~0.6 mmol/kg dissolved CO_2_). The peridotite was sampled from Panshishan (Jiangsu Province, China) where it occurs as xenoliths in basalt^57^,^58^. It is composed of 60–65% olivine, 20–25% orthopyroxene, 15% clinopyroxene, and 1–3% spinel. The sample was crushed and sieved into grain sizes of <30, 42–59, and 100–177 μm.

All experiments were conducted in the high-pressure and high-temperature laboratory at Guangzhou Institute of Geochemistry, Chinese Academy of Sciences. Experimental procedures were essentially the same as those described in another experimental study^40^. The reactants and starting fluid were sealed into gold capsules, which were placed into the end of hydrothermal vessels, followed with a filler rod. After heating, the vessels were quenched to room temperature in cold water within 10 min.

The gas components in the gold capsules were analysed using an Agilent 7890A gas chromatograph at the State Key Laboratory of Organic Geochemistry, Guangzhou Institute of Geochemistry. The gold capsule was placed in a vacuum glass piercer, which was connected to a Toepler pump and a volume-calibrated glass pipe through vacuum line. The gold capsule was pierced by a steel needle in vacuum (with a pressure of less than 1 × 10^−2^ Pa), and all of gas components were concentrated by a Toepler pump into the volume-calibrated pipe. The hydrocarbons were quantified using an external standard with an accuracy of less than 0.5%. The detailed analysis procedures have been reported in previous studies^40^,^59^,^60^.

After gas chromatography analyses, the remaining gas in the vacuum glass piercer and glass pipe, with an amount about 80% of the initial value, was taken with a syringe for gas chromatography-isotope ratio mass spectrometry analyses. The carbon isotope value of CO_2_ reference gas was calibrated by NBS 22 oil as a reference using element analysis, combined with isotope ratio mass spectrum. Carbon isotope values of methane were calculated with CO_2_ as a reference gas that was automatically loaded into the system at the beginning and the end of each analysis.

The surface morphology of solid products was characterized with a Zeiss Ultra 55 Field emission gun scanning electron microscope at Second Institute of Oceanography, State Oceanic Administration of China. Fourier transformed infrared spectroscopy analyses were performed using a Bruker Vector 33 FTIR spectrometer at Analytical and Testing Center of South China University of Technology. Infrared spectra were obtained at wavenumbers from 400 to 4000 cm^−1^ at a resolution of 4 cm^−1^ with 32 scans for each spectrum. The KBr pellets were prepared by mixing around 1 mg of sample powder with 200 mg of KBr.

## Additional Information

**How to cite this article**: Huang, R. *et al*. The H_2_/CH_4_ ratio during serpentinization cannot reliably identify biological signatures. *Sci. Rep.* **6**, 33821; doi: 10.1038/srep33821 (2016).

## Figures and Tables

**Table 1:** Experimental conditions and gas compositions.

Sample No.	T (°C)	P (kbar)	Time (days)	Initial grain sizes (μm)	W/R ratio	H_2_ (m)	CH_4_ (m)	C_2_H_6_ (μ)	C_3_H_8_ (μ)	H_2_/CH_4_	δ^13^CH_4_ (vs. PDB)
HR61	311	3.1	120	100–177	0.82	127	0.06	2.72	5.44	2,120	
HR77	311	3.0	28	100–177	1.2	10.1	0.028	26.5	39.5	360	
HR86	311	3.0	27	<30	0.86	119	1.3	59.3	316	91	
HR91	311	3.0	14	<30	0.89	167	2.88	161	322	58	
HR105	311	3.0	10	<30	1.0	21	0.35	23.6	9.2	60	
HR68	400	3.0	20	<30	0.71	2.3	11.8	1,686	1,384	0.2	
HR78	400	3.0	29	100–177	1.0	3	0.37	36.9	35.4	8.2	
HR79	400	3.0	27	100–177	0.86	1.3	0.37	4.0	0	3.5	
HR90	400	3.0	24	100–177	0.93	2.3	3.8	526	129	0.6	
HR98	400	3.2	31	42–59	0.96	5.6	13.2	591	214	0.4	
HR102	400	3.0	38	42–59	1.1	0.4	4.1	208	40.1	0.1	−24
HR70	500	3.0	20	<30	0.98	13.5	15.6	24.4	61.8	0.9	
HR82	500	3.0	36	<30	1.0	2.8	48.8	34.1	6.66	0.05	
HR81	500	3.3	22	100–177	0.88	0.8	0.51	21.3	0.01	1.6	
HR101	500	3.2	9	<30	0.71	3.3	15.0	110	14.0	0.2	
HR103	500	3.0	15	<30	0.61	1.2	7.2	9.9	4.97	0.2	−22

W/R ratio: ratio between the mass of the starting fluids and solid materials loaded in gold capsules.

The starting materials for HR101 and HR103 are mechanical mixtures of olivine and SiO_2_ with a mass ratio of 1:1.

## References

[b1] Charlou J. L. . Mineral and gas chemistry of hydrothermal fluids on an ultrafast spreading ridge: East Pacific rise, 17° to 19°S (Naudur cruise, 1993) phase separation processes controlled by volcanic and tectonic activity. J. Geophys. Res. 101, 15899–15919 (1996).

[b2] Charlou J. L. . Intense CH_4_ plumes generated by serpentinization of ultramafic rocks at the intersection of the 15°20′N fracture zone and the Mid-Atlantic Ridge. Geochim. Cosmochim. Acta 62(13), 2323–2333 (1998).

[b3] Charlou J. L., Donval J. P., Fouquet Y., Jean-Baptiste P. & Holm N. Geochemistry of high H_2_ and CH_4_ vent fluids issuing from ultramafic rocks at the Rainbow hydrothermal field (36°14′N, MAR). Chem. Geol. 191, 345–359 (2002).

[b4] Hyndman R. D. & Peacock S. M. Serpentinization of the forearc mantle. Earth Planet Sci. Lett. 212, 417–432 (2003).

[b5] Mével C. Serpentinization of abyssal peridotites at mid-ocean ridges. C. R. Geosci. 335, 825–852 (2003).

[b6] Evans B. W., Hattori K. & Baronnet A. Serpentinite: What, why, where? Elements 9, 99–106 (2013).

[b7] Ehlmann B. L. . Orbital identification of carbonate-bearing rocks on Mars. Science 322, 1828–1832 (2008). 19095939 10.1126/science.1164759

[b8] Ehlmann B. L. . Identification of hydrated silicate minerals on Mars using MRO-CRISM: Geologic context near Nili Fossae and implications for aqueous alteration. J. Geophys. Res. 114, E00D08 (2009).

[b9] Ehlmann B. L., Mustard J. F. & Murchie S. L. Geologic setting of serpentine deposits on Mars. Geophys. Res. Lett. 37, L06201 (2010).

[b10] Escartín J., Hirth G. & Evans B. Strength of slightly serpentinized peridotites: implications for the tectonics of oceanic lithosphere. Geology 29, 1023–1026 (2001).

[b11] Scambelluri M., Rampone E. & Piccardo G. B. Fluid and element cycling in subducted serpentinite: A trace-element study of the Erro-Tobbio high-pressure ultramafites (western Alps, NW Italy). J. Petrol. 42(1), 55–67 (2001).

[b12] Scambelluri M., Fiebig J., Malaspina N., Müntener O. & Pettke T. Serpentinite subduction: Implications for fluid processes and trace-element recycling. Int. Geol. Rev. 46, 595–613 (2004).

[b13] Scambelluri M., Pettke T., Rampone E., Godard M. & Reusser E. Petrology and trace element budgets of high-pressure peridotite indicate subduction dehydration of serpentinized mantle (Cima di Gagnone, central Alps, Switzerland). J. Petrol. 55, 459–498 (2014).

[b14] Hattori K. H. & Guillot S. Volcanic fronts as a consequence of serpentinites dehydration in the fore-arc mantle wedge. Geology 31, 525–528 (2003).

[b15] Guillot S. & Hattori K. Serpentinites: Essential roles in geodynamics, arc volcanism, sustainable development, and the origin of life. Elements 9, 95–98 (2013).

[b16] Scambelluri M., Müntener O., Hermann J., Piccardo G. B. & Trommosdorff V. Subduction of water into the mantle: History of an Alpine peridotite. Geology 23, 459–462 (1995).

[b17] Deschamps F. . Behavior of fluid-mobile elements in serpentinites from abyssal to subduction environments: Examples from Cuba and Dominican Republic. Chem. Geol. 312–313, 93–117 (2012).

[b18] Ulmer P. & Trommsdorff V. Serpentine stability to mantle depths and subduction-related magmatism. Science 268, 858–861 (1995). 17792181 10.1126/science.268.5212.858

[b19] Schmidt M. W. & Poli S. Experimentally based water budgets for hydrating slabs and consequences for arc magma generation. Earth Planet Sci. Lett. 163, 361–379 (1998).

[b20] Kelley D. S. . An off-axis hydrothermal vent field near the Mid-Atlantic Ridge at 30°N. Nature 412, 145–149 (2001). 11449263 10.1038/35084000

[b21] Holm N. G. & Charlou J. L. Initial indications of abiotic formation of hydrocarbons in the Rainbow ultramafic hydrothermal system, Mid-Atlantic Ridge. Earth Planet Sci. Lett. 191, 1–8 (2001).

[b22] Schrenk M. O., Kelley D. S., Bolton S. A. & Baross J. A. Low archaeal diversity linked to subseafloor geochemical processes at the Lost City Hydrothermal Field, Mid-Atlantic Ridge. Environ. Microbiol. 6(10), 1086–1095 (2004). 15344934 10.1111/j.1462-2920.2004.00650.x

[b23] Lang S. Q., Butterfield D. A., Schulte M., Kelly D. S. & Lilley M. D. Elevated concentrations of formate, acetate and dissolved organic carbon found at the Lost City hydrothermal field. Geochim. Cosmochim. Acta 74, 941–952 (2010).

[b24] Brazelton W. J., Schrenk M. O., Kelley D. S. & Baross J. A. Methane- and sulfur-metabolizing microbial communities dominate the Lost City Hydrothermal Field ecosystem. Appl. Environ. Microbiol. 72(9), 6257–6270 (2006). 16957253 10.1128/AEM.00574-06 PMC1563643

[b25] Brazelton W. J., Nelson B. & Schrenk M. O. Metagenomic evidence for H_2_ oxidation and H_2_ production by serpentinite-hosted subsurface microbial communities. Front. Microbiol . 2, 1–16 (2012). 10.3389/fmicb.2011.00268 PMC3252642 22232619

[b26] Schrenk M. O., Brazelton W. J. & Lang S. Q. Serpentinization, carbon, and deep life. Rev. Mineral. Geochem. 75, 575–606 (2013).

[b27] Oze C., Jones L. C., Goldsmith J. I. & Rosenbauer R. J. Differentiating biotic from abiotic methane genesis in hydrothermally active planetary surfaces. Proc. Natl. Acad. Sci. USA 109, 9750–9754 (2012). 22679287 10.1073/pnas.1205223109 PMC3382529

[b28] Bradley A. S. & Summons R. E. Multiple origins of methane at the Lost City hydrothermal field. Earth Planet Sci. Lett. 297, 34–41 (2010).

[b29] Horita J. & Berndt M. E. Abogenic methane formation and isotopic fractionation under hydrothermal conditions. Science 285, 1055–1057 (1999). 10446049 10.1126/science.285.5430.1055

[b30] McCollom T. M. & Seewald J. S. Carbon isotope composition of organic compounds produced by abiotic synthesis under hydrothermal conditions. Earth Planet Sci. Lett. 243, 74–84 (2006).

[b31] Berndt M. E., Allen D. E. & Seyfried W. E. Jr. Reduction of CO_2_ during serpentinization of olivine at 300 °C and 500 bar. Geology 24(4), 351–354 (1996).

[b32] Allen D. E. & Seyfried W. E. Jr. Compositional controls on vent fluids from ultramafic-hosted hydrothermal systems at mid-ocean ridges: An experimental study at 400 °C, 500 bars. Geochim. Cosmochim. Acta 67(8), 1531–1542 (2003).

[b33] McCollom T. M. & Bach W. Thermodynamic constraints on hydrogen generation during serpentinization of ultramafic rocks. Geochim. Cosmochim. Acta 73, 865–875 (2009).

[b34] Seyfried W. E. Jr., Foustoukos D. I. & Fu Q. Redox evolution and mass transfer during serpentinization: An experimental and theoretical study at 200 °C, 500 bar with implications for ultramafic-hosted hydrothermal systems at mid-ocean ridges. Geochim. Cosmochim. Acta 71, 3872–3886 (2007).

[b35] Marcaillou C., Muñoz M., Vidal O., Parra T. & Harfouche M. Mineralogical evidence for H_2_ degassing during serpentinization at 300 °C/300 bar. Earth Planet Sci. Lett. 303, 281–290 (2011).

[b36] Lazar C., McCollom T. M. & Manning C. E. Abiogenic methanogenesis during experimental komatiite serpentinization: Implications for the evolution of the early Precambrian atmosphere. Chem. Geol. 326–327, 102–112 (2012).

[b37] Foresti E. . Determination of low levels of free fibres of chrysotile in contaminated soils by X-ray diffraction and FTIR spectroscopy. Anal. Bioanal. Chem. 376, 653–658 (2003). 12802568 10.1007/s00216-003-1965-3

[b38] Anbalagan G., Sivakumar G., Prabakaran A. R. & Gunasekaran S. Spectroscopic characterization of natural chrysotile. Vib. Spectrosc. 52, 122–127 (2010).

[b39] Lafay R. . Simultaneous precipitation of magnesite and lizardite from hydrothermal alteration of olivine under high-carbonate alkalinity. Chem. Geol. 368, 63–75 (2014).

[b40] Huang R. F., Sun W. D., Ding X., Liu J. Z. & Peng S. B. Olivine versus peridotite during serpentinization: Gas formation. Sci. China Earth Sci. 58(12), 2165–2174 (2015).

[b41] Bonifacie M. . Chlorine isotopic composition in seafloor serpentinites and high-pressure metaperidotites. Insights into oceanic serpentinization and subduction processes. Geochim. Cosmochim. Acta 72, 126–139 (2008).

[b42] Liu X. W., Liu X. X. & Hu Y. H. Investigation of the thermal decomposition of talc. Clay Clay Miner . 62(2), 137–144 (2014).

[b43] Tingle T. N., Hochella M. F. Jr., Becker C. H. & Malhotra R. Organic compounds on crack surfaces in olivine from San Carlos, Arizona, and Hualalai Volcano, Hawaii. Geochim. Cosmochim. Acta 54, 477–485 (1990).

[b44] Sugisaki R. & Mimura K. Mantle hydrocarbons: Abiotic or biotic? Geochim. Cosmochim. Acta 58, 2527–2542 (1994). 11541663 10.1016/0016-7037(94)90029-9

[b45] McCollom T. M. & Seewald J. S. A reassessment of the potential for reduction of dissolved CO_2_ to hydrocarbons during serpentinization of olivine. Geochim. Cosmochim. Acta 65(21), 3769–3778 (2001).

[b46] Martin B. & Fyfe W. S. Some experimental and theoretical observations on the kinetics of hydration reactions with particular reference to serpentinization. Chem. Geol. 6, 185–202 (1970).

[b47] Wegner W. W. & Ernst W. G. Experimentally determined hydration and dehydration reaction rates in the system MgO-SiO_2_-H_2_O. Am. J. Sci. 283-A, 151–180 (1983).

[b48] Malvoisin B., Brunet F., Carlut J., Rouméjon S. & Cannat M. Serpentinization of oceanic peridotites: 2. Kinetics and progresses of San Carlos olivine hydrothermal alteration. J. Geophys. Res. 117, B04102 (2015).

[b49] Seewald J. S. & Seyfried W. E. Jr. The effect of temperature on metal mobility in subseafloor hydrothermal systems: Constraints from basalt alteration experiments. Earth Planet Sci. Lett. 101, 388–403 (1990).

[b50] Douville E. . The rainbow vent fluids (36°14′N, MAR): The influence of ultramafic rocks and phase separation on trace metal content in Mid-Atlantic Ridge hydrothermal fluids. Chem. Geol. 184, 37–48 (2002).

[b51] Frost B. R. & Beard J. S. On silica activity and serpentinization. J. Petrol. 48(7), 1351–1368 (2007).

[b52] Seyfried W. E. Jr., Pester N. J., Ding K. & Rough M. Vent fluid chemistry of the Rainbow hydrothermal system (36°N, MAR): Phase equilibria and *in situ* pH controls on subseafloor alteration processes. Geochim. Cosmochim. Acta 75, 1574–1593 (2011).

[b53] Huang R. F., Sun W. D., Ding X., Wang Y. R. & Zhan W. H. Experimental study on the formation of hydrogen gas and methane during serpentinization. Acta Petrol. Sin. 31(7), 1901–1907 (2015).

[b54] Lang S. Q. . H_2_/CH_4_ ratios cannot reliably distinguish abiotic vs. biotic methane in natural hydrothermal systems. Proc. Natl. Acad. Sci. USA 109(47), E3210 (2012). 23012469 10.1073/pnas.1213138109 PMC3511145

[b55] Proskurowski G. . Abiogenic hydrocarbon production at Lost City hydrothermal field. Science 319, 604–607 (2008). 18239121 10.1126/science.1151194

[b56] Fu Q. . Abiotic formation of hydrocarbons under hydrothermal conditions: Constraints from chemical and isotope data. Geochim. Cosmochim. Acta 71, 1982–1998 (2007).

[b57] Chen D. G., Li B. X. & Zhi X. C. Genetic geochemistry of mantle-derived peridotite xenolith from Panshishan, Jiangsu. Geochimica 23, 13–24 (1994).

[b58] Xu X. S. . Re-Os isotopes of sulfides in mantle xenoliths from eastern China: Progressive modification of lithospheric mantle. Lithos 102, 43–64 (2008).

[b59] Xiong Y. . Kinetic simulating experiment on the secondary hydrocarbon generation of kerogen. Sci China (Ser D) 45, 13–20 (2001).

[b60] Pan C. C., Yu L. P., Liu J. Z. & Fu J. M. Chemical and carbon isotopic fractionations of gaseous hydrocarbons during abiogenic oxidation. Earth Planet Sci. Lett. 246, 70–89 (2006).

